# Effectiveness of Demonstration-Observation-Assistance-Performance (DOAP) Versus Video-Assisted Learning (VAL) for Obstetric Examination Skills Among Phase 3 Bachelor of Medicine and Bachelor of Surgery (MBBS) Students

**DOI:** 10.7759/cureus.98321

**Published:** 2025-12-02

**Authors:** HU Bhavya, Y Vipulachandra, Mahesh Babu

**Affiliations:** 1 Obstetrics and Gynecology, Subbaiah Institute of medical Sciences, Shivamogga, IND; 2 Gynecologic Oncology, Mazumdar Shaw Medical college, Narayana Health City, Bangalore, IND; 3 Ophthalmology, Subbaiah Institute of Medical Sciences, Shivamogga, IND

**Keywords:** blended learning, cbme, clinical skills, doap, indian medical graduate, medical education, obstetric examination, phase 3 mbbs students, retention of skills, video-assisted learning

## Abstract

Background: Bedside teaching is vital for cultivating essential antenatal exam skills that many graduates lack despite their theoretical knowledge. Methods like video-assisted learning (VAL) and demonstration-observation-assistance-performance (DOAP) bring a visual, interactive edge to skill acquisition, though studies show mixed results on which method is superior. This study compares the effectiveness of VAL versus DOAP and students' perceptions of teaching obstetric examination skills to phase 3 part 2 Bachelor of Medicine and Bachelor of Surgery (MBBS) students.

Materials and methods: This prospective interventional study was conducted among 60 phase 3 part 2 MBBS students in the department of obstetrics and gynecology. Students were assigned to DOAP or VAL groups by systematic sampling (alternate roll numbers), with a crossover after three sessions covering six obstetric examination competencies. Assessment was done on the next day (day one) and on day 15 following each session using an objective structured clinical examination (OSCE) checklist. An unpaired t‑test compared VAL versus DOAP at both assessments; paired t‑tests evaluated skill retention by comparing day one and day 15 scores within each method.

Results: Total score analysis shows that students in DOAP had higher mean scores of 24.40 ± 2.98 and 21.80 ± 2.25 than those in VAL, 21.37 ± 3.52 and 18.93 ± 2.92, for the first and second assessments, respectively. The findings of our study assessing retention in the second assessment showed that DOAP generally resulted in higher mean scores across all sessions than VAL. DOAP is perceived as more satisfying, effective, confidence-boosting, recommendable, and engaging.

Conclusions: While both DOAP and VAL are effective for teaching obstetric examination skills, the DOAP method may offer additional benefits in terms of skill performance and student confidence. In contrast, while VAL offers the advantages of flexibility and visual reinforcement, it may not fully replicate the interactive and practical experiences provided by DOAP. We should consider combining both methods to maximize learning outcomes for phase 3 MBBS students.

## Introduction

The backbone of undergraduate training in obstetrics and gynecology remains bedside teaching. However, studies report inadequate graduate proficiency in examination skills, communication, and doctor-patient relationships, leading to suboptimal antenatal care [1-4]. With India’s shift to competency-based medical education, Indian medical graduates (IMGs) must master clinical skills, such as the antenatal examination, which are crucial for diagnosis and fetal assessment [4,5]. IMGs will be the first to come into contact with patients, especially in rural areas of India. They need to recognize, triage, and refer the patient in a timely manner when required. However, conventional methods yield wide variability in bedside obstetric examination skills among IMGs [5,6].

Structured approaches like video-assisted learning (VAL) add visual, interactive elements to traditional teaching, enhancing engagement and retention [7-10]. The demonstration-observation-assistance-performance (DOAP) framework sequentially guides students from observation to independent practice [11]. Studies comparing DOAP and VAL show mixed results in skill acquisition and student preference [12-16]. Hence, this study was done to determine and compare the effectiveness of DOAP and VAL as a teaching-learning method for obstetric examination skills among phase 3 part 2 Bachelor of Medicine and Bachelor of Surgery (MBBS) students, and also to assess the perception of students toward the use of VAL and DOAP as a teaching-learning method in obstetric skills.

## Materials and methods

This quasi-experimental, prospective, interventional study was conducted from March to June 2024 among 60 consenting phase 3 part 2 MBBS students posted in the Department of Obstetrics and Gynecology at a teaching institute, following approval from the K. V. G. Medical College and Hospital Institutional Ethics Committee (approval number: KVGMCIEC202467). A convenient sampling technique was applied. Students absent during teaching or assessment days were excluded. Thirty participants were assigned to Group A and Group B using a systematic sampling technique (alternate roll numbers were assigned to each group). Two teaching methods, DOAP and VAL, were used, and outcomes were measured with objective structured clinical examination (OSCE) checklists and a Likert-scale feedback questionnaire.

Training comprised six competencies delivered across six sessions, one per session: obtaining informed consent for obstetric examination (session 1), ensuring prerequisites for examination (session 2), demonstrating inspection findings (session 3), demonstrating palpation techniques (session 4), demonstrating obstetric grips (session 5), auscultating fetal heart sounds, and summarizing findings (session 6).

During the first three alternate-day sessions (10-15 minutes each), Group A received DOAP instruction from experienced obstetrics and gynecology faculty: after observing a demonstration, students described the procedure stepwise, assisted in examination, and performed it on patients under supervision. Group B watched video demonstrations by the same faculty and then practiced the skills. After these sessions, the groups crossed over for the remaining three competencies, with equivalent time allocation. Each session was done for both groups on the same day (day 0), and the assessment was done on the next day (day 1) and on day 15 of the session using the OSCE checklist. The same method was followed after the groups crossed over.

**Figure 1:**
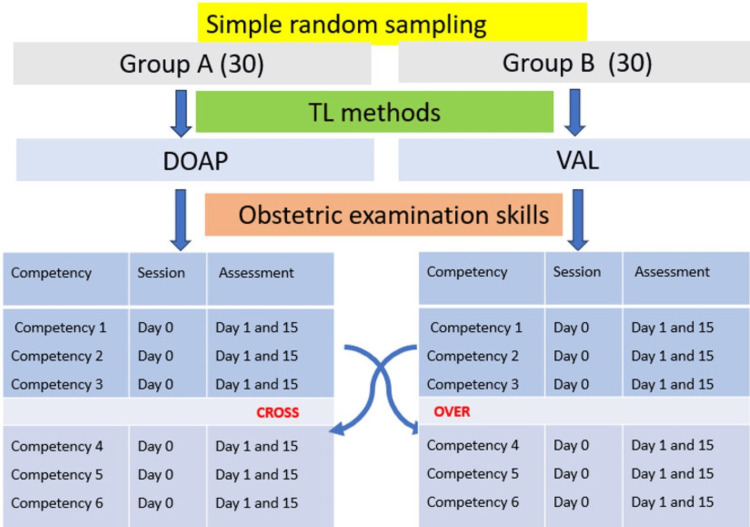
Schematic representation of data collection DOAP: demonstration-observation-assistance-performance, VAL: video-assisted learning

Checklists, developed by departmental faculty and the Medical Education Unit, contained five items each, scored 1 for correct and 0 for incorrect performance (a maximum of 5 per checklist, with a total possible score of 30). Schematic representation of data collection is shown in Figure 1.

Mean scores ± SD were calculated for each session and method. An unpaired t‑test compared VAL versus DOAP at both assessments; paired t‑tests evaluated skill retention by comparing day one and day 15 scores within each method. A p‑value of <0.05 denotes statistical significance.

At the study's end, students completed the Google Forms questionnaire (Mountain View, CA, USA) with five-point Likert-scale items to capture perceptions of VAL and DOAP. Remedial training was provided to low-performing students before the completion of clinical posting. To maintain ethical equity, remaining sessions employed the alternate teaching method after the study’s conclusion.

## Results

This study includes data from 60 participants who were present on the day of the teaching session and on the assessment days. The participants ranged in age from 22 to 24 years, with a mean age of 23.05 ± 0.59 years. Among 60 participants, 36 were girls (60%), and the remaining 24 (40%) were boys.

Table 1 represents the results of a comparison between two groups (A and B) using the DOAP and VAL across multiple sessions to teach obstetric examination skills. During the first assessment, mean scores were consistently higher in DOAP than in VAL for both groups A and B across most sessions, with significant differences observed in sessions II, IV, V, and VI. Overall, it shows that students in DOAP had a higher mean score (24.40 ± 2.98) than those in VAL (21.37 ± 3.52), even after crossover was performed among groups.

**Table 1:** Comparison of DOAP and VAL group scores after each session on day 1 (first assessment) using the unpaired t-test DOAP: demonstration-observation-assistance-performance, VAL: video-assisted learning, SD: standard deviation, CI: confidence interval

Sessions	Group	DOAP (mean score ± SD)	95% CI	Group	VAL (mean score ± SD)	95% CI	t-value	p-value
I (informed consent)	A	4.76 ± 0.44	4.60 to 4.92	B	4.53 ± 0.51	4.34 to 4.72	1.87	0.055
II (​​​prerequisites for examination)		3.62 ± 0.86	3.30 to 3.94		3.10 ± 0.84	2.79 to 3.41	2.37	0.011
III (inspection)		3.72 ± 0.92	3.38 to 4.06		3.50 ± 0.82	3.19 to 3.81	0.98	0.163
IV (palpation)	B	4.24 ± 0.69	3.98 to 4.50	A	3.60 ± 1.25	3.13 to 4.07	2.46	0.009
V (obstetric grips)		4.17 ±1.00	3.80 to 4.54		3.47 ± 1.33	2.97 to 3.97	2.30	0.012
VI (auscultation and summarize)		4.21 ± 1.95	3.48 to 4.94		3.20 ± 1.24	2.74 to 3.66	2.39	0.004
Total mean score		24.40 ± 2.98	23.29 to 25.51		21.37 ± 3.52	20.06 to 22.68	3.6	<0.001

Mean scores obtained for each session in the second assessment among groups A and B for both DOAP and VAL are given in Table 2. Assessment scores for the first three contact sessions for groups A and B (I, II, and III) in both teaching methods, DOAP and VAL, were almost identical and not statistically significant, indicating similar effectiveness of both methods in these sessions. Students from group B scored better in the last three sessions than those in group A, who had VAL during those sessions. Mean scores for sessions IV, V, and VI showed a significant difference between DOAP and VAL, in which students in DOAP (group B) had scored better (4.69 ± 0.47, 3.86 ± 0.67, and 3.24 ± 0.44) than those in VAL (group A), whose mean scores were 3.80 ± 0.92, 2.77 ± 0.94, and 2.23 ± 0.73, respectively, with a p-value of <0.001. The overall mean score for the second assessment, conducted 15 days after the first, was 21.80 for DOAP and 18.93 ± 2.92 for VAL. The difference was found to be statistically significant (p<0.001). On comparing, the overall scores of the second assessment were lower than those of the first assessment in both DOAP and VAL.

**Table 2:** Comparison of DOAP and VAL group scores after each session on day 15 (second assessment) using the unpaired t-test DOAP: demonstration-observation-assistance-performance, VAL: video-assisted learning, SD: standard deviation, CI: confidence interval

Sessions	Group	DOAP (mean score ± SD)	95% CI	Group	VAL (mean score ± SD)	95% CI	t-value	p-value
I (informed consent)	A	3.76 ± 0.44	3.60 to 3.92	B	3.57 ± 0.68	3.32 to 3.82	1.28	0.133
II (​​​prerequisites for examination)		3.87 ± 0.68	3.62 to 4.12		4.00 ± 0.79	3.71 to 4.29	-0.68	0.243
III (inspection)		2.72 ± 0.92	2.38 to 3.06		2.56 ± 0.68	2.31 to 2.81	0.76	0.229
IV (palpation)	B	4.69 ± 0.47	4.51 to 4.87	A	3.80 ± 0.92	3.46 to 4.14	4.71	<0.001
V (obstetric grips)		3.86 ± 0.67	3.61 to 4.11		2.77 ± 0.94	2.42 to 3.12	5.17	<0.001
VI (auscultation and summarize)		3.24 ± 0.44	3.08 to 3.40		2.23 ± 0.73	1.96 to 2.50	6.49	<0.001
Total mean score		21.80 ± 2.25	20.96 to 22.64		18.93 ± 2.92	17.84 to 20.02	4.26	<0.001

The comparison of the first and second assessment scores for students in groups A and B exposed to DOAP across all six sessions is shown in Table 3. The scores of the second assessment for the sessions I, III, and VI (4.76 ± 0.44, 3.72 ± 0.92, and 4.21 ± 1.95) were found to be less than the scores of the first assessment, and the difference was found to be statistically significant (p<0.001). But scores for session IV were slightly higher in the second assessment (4.6 ± 0.47) than in the first, although it was not statistically significant. Overall, there was a statistically significant (p<0.001) decrease in scores from the first to the second assessment across all sessions combined, with mean scores of 24.4 ± 2.97 and 21.80 ± 2.25, respectively.

**Table 3:** Comparing scores between the first and second assessment among students exposed to DOAP by paired t-test DOAP: demonstration-observation-assistance-performance, SD: standard deviation, CI: confidence interval

Sessions	DOAP				t-value	p-value
	First assessment (mean ± SD)	95% CI	Second assessment (mean ± SD)	95% CI		
I (informed consent)	4.76 ± 0.44	4.60 to 4.92	3.76 ± 0.44	3.60 to 3.92	12.44	<0.001
II (​​​prerequisites for examination)	3.62 ± 0.86	3.30 to 3.94	3.87 ± 0.68	3.62 to 4.12	-1.74	0.056
III (inspection)	3.72 ± 0.92	3.38 to 4.06	2.70 ± 0.90	2.37 to 3.03	6.13	<0.001
IV (palpation)	4.24 ± 0.69	3.98 to 4.50	4.69 ± 0.47	4.52 to 4.86	-4.03	0.412
V (obstetric grips)	4.17 ± 1.00	3.80 to 4.54	3.86 ± 0.67	3.61 to 4.11	1.92	0.142
VI (auscultation and summarize)	4.21 ± 1.95	3.48 to 4.94	3.24 ± 0.44	3.08 to 3.40	2.99	<0.001
Total mean score	24.40 ± 2.98	23.29 to 25.51	21.80 ± 2.25	20.96 to 22.64	5.29	<0.001

The results presented in Table 4 show the comparison of scores between the first and second assessments among students who were exposed to VAL. Second assessment scores for most of the sessions, like I, III, V, and VI (3.57 ± 0.68, 2.56 ± 0.68, 2.77 ± 0.94, and 2.23 ± 0.73), show a significant decrease, suggesting a decline in performance. In contrast, scores for session II show a significant increase (4.00 ± 0.79), indicating improved performance, and session IV shows no significant change (3.80 ± 0.92), indicating consistent performance. The overall mean score significantly declined from the first assessment (21.37 ± 3.52) to the second assessment (18.93 ± 2.92), suggesting a decline in performance across sessions among students exposed to VAL.

**Table 4:** Comparing scores between first and second assessment among students exposed to VAL by paired t-test VAL: video assisted learning, SD: standard deviation, CI: confidence interval

Sessions	VAL				t-value	p-value
	First assessment (mean ± SD)	95% CI	Second assessment (mean ± SD)	95% CI		
I (informed consent)	4.53 ± 0.51	4.34 to 4.72	3.57 ± 0.68	3.32 to 3.82	4.96	<0.001
II (​​​prerequisites for examination)	3.10 ± 0.84	2.78 to 3.42	4.00 ± 0.79	3.71 to 4.29	2.57	<0.001
III (inspection)	3.50 ± 0.82	3.19 to 3.81	2.56 ± 0.68	2.31 to 2.81	5.31	<0.001
IV (palpation)	3.60 ± 1.25	3.13 to 4.07	3.80 ± 0.92	3.46 to 4.14	0.7	0.396
V (obstetric grips)	3.47 ± 1.33	2.98 to 3.96	2.77 ± 0.94	2.43 to 3.11	2.4	0.022
VI (auscultation and summarize)	3.20 ± 1.24	2.73 to 3.67	2.23 ± 0.73	1.96 to 2.50	5.35	<0.001
Total score	21.37 ± 3.52	20.05 to 22.69	18.93 ± 2.92	17.84 to 20.02	7.4	<0.001

**Figure 2:**
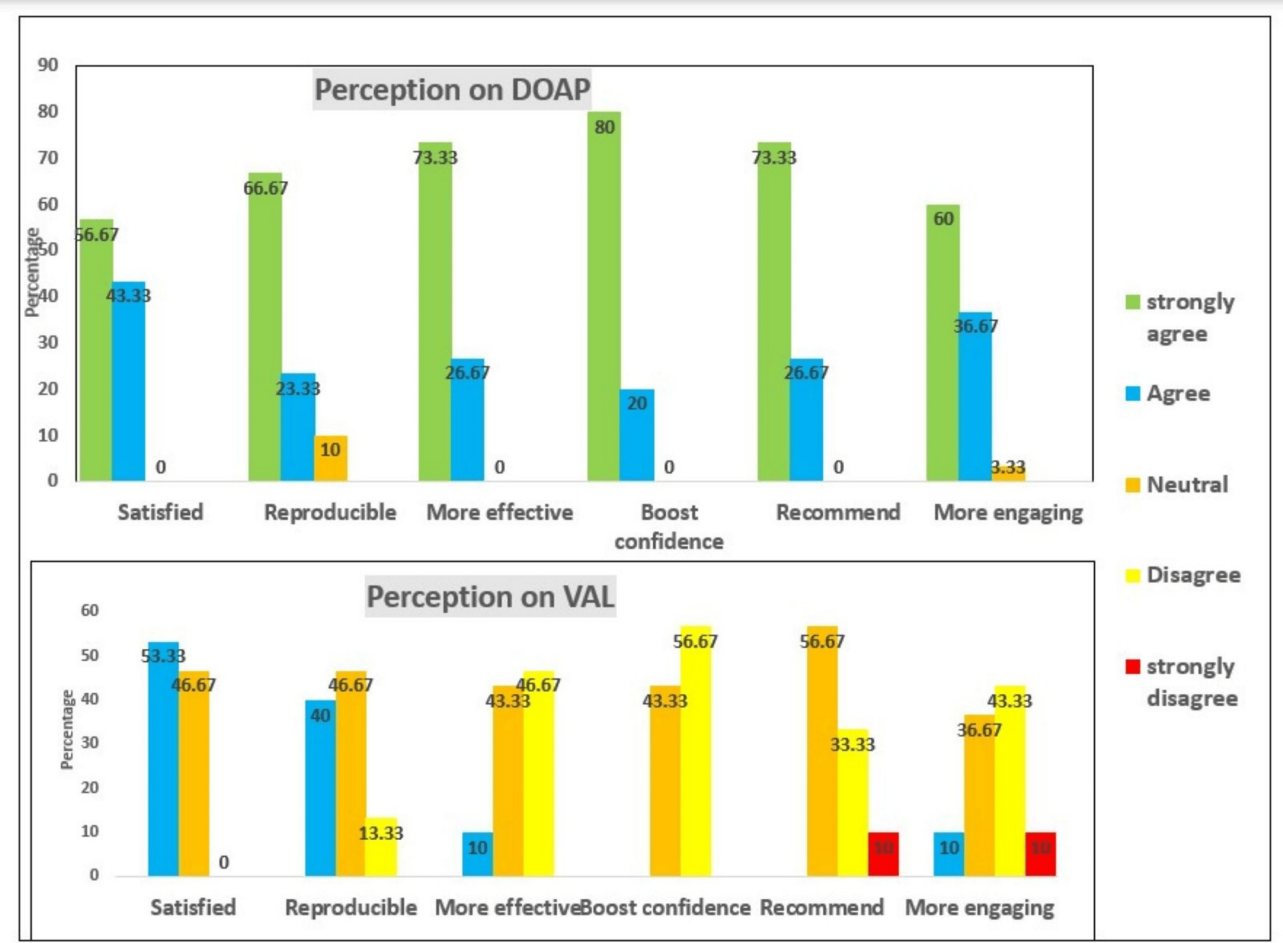
Perception of the students for DOAP and VAL as teaching-learning method for obstetric examination skills DOAP: demonstration-observation-assistance-performance, VAL: video-assisted learning

Figure 2 presents students' responses to various parameters, indicating their level of agreement with each statement regarding the TL methods, DOAP, and VAL. The majority of students were generally satisfied with the DOAP method, with 56.67% strongly agreeing. VAL also shows a reasonable level of satisfaction, though with no strong agreement and a neutral response rate of 46.67%.

DOAP is perceived as more helpful for reproducibility, with a majority strongly agreeing (66.67%). VAL shows weaker agreement, greater neutrality (46.67%), and greater disagreement. DOAP is considered highly effective for learning obstetric examination skills, with 73.33% of students strongly agreeing. In contrast, VAL is seen as less effective, with a large proportion of students disagreeing (46.67%) or remaining neutral (43.33%). DOAP significantly boosts students' confidence, with almost all respondents strongly agreeing (80%). VAL does not seem to improve confidence as effectively, with a majority disagreeing (56.67%). Students are highly likely to recommend DOAP to healthcare professionals. VAL has a lower recommendation rate, with many students neutral (56.67%). DOAP is viewed as more engaging and easier to follow, with strong agreement (60%). VAL, however, is seen as less engaging, with significant neutrality and disagreement (43.33%).

Overall, students' perception strongly favors DOAP over VAL. DOAP is perceived as more satisfying, effective, confidence-boosting, recommendable, and engaging. In contrast, VAL receives more neutral or negative responses, despite a few positive perceptions.

## Discussion

This interventional study, conducted in the Department of Obstetrics and Gynecology at a tertiary care teaching institute, aimed to compare the effectiveness of DOAP versus VAL in teaching obstetric examination skills to phase 3 part 2 MBBS students. This comprehensive study provides an insight into the impact of two different teaching methods on the acquisition of obstetric examination skills. The study utilized a rigorous assessment method involving OSCE checklists developed with faculty consensus. The immediate and two-week follow-up assessments provided robust measures of short-term and long-term skill retention, respectively. The crossover between groups A and B, in which students experienced both teaching methods, enabled a fair comparison and minimized bias. By maintaining equal time allocation for both methods and conducting assessments at consistent intervals, the study ensured that differences in student performance could be attributed to the teaching methods rather than external variables.

Initial assessment results in our study showed that students who were exposed to DOAP as a teaching method for learning obstetric examination skills in both groups A and B performed better, with higher scores, than those in VAL. This suggests that DOAP provides a more effective learning experience for obstetric examination skills, likely because of its hands-on, interactive nature. While VAL also contributed to student learning, it was generally less effective than DOAP, as indicated by the lower mean scores. VAL's benefits might lie in providing visual and repetitive learning opportunities, which can be especially beneficial when used alongside hands-on practice.

A study by Madavan on advanced cardiac life support training using DOAP and VAL showed that learners performed better with DOAP. However, both methods were equally effective in imparting knowledge, skills, and attitude, with students providing positive feedback for DOAP [12]. A study by Devi et al. also found that direct demonstration scored much better than video-assisted teaching for antenatal skills and concluded that demonstration is more effective in developing skills, suggesting that we can enhance teaching skills by adopting a blended teaching approach to improve retention and reproducibility [16]. The outcomes of these studies were consistent with our current study's findings regarding the effectiveness of DOAP and VAL as teaching methods.

In contrast, a study by Shirly Kurian and Gokul found that assessment scores were higher in the video-assisted group than in the traditional bedside teaching group. It concluded that a blended teaching approach can be adopted by providing a bedside demonstration of skills, which is then reinforced by a video demonstration [15]. Additionally, a study by Padmavathi et al. regarding video demonstration as a teaching-learning method for a core clinical skill among undergraduate medical students showed better performance in the video-assisted teaching group [8].

The findings of our study assessing retention in the second assessment showed that DOAP generally resulted in higher mean scores across all sessions than VAL, with statistically significant differences observed in sessions IV, V, and VI. While VAL was comparable to DOAP in the initial sessions, which involved taking consent and arranging perquisites for examination (I, II, and III), it was less effective in later sessions, which involved the demonstration of obstetric examinations, such as measurement of symphysio-fundal height, grips, and localizing fetal heart sounds. This suggests that retention was similar across the first three sessions in both DOAP and VAL, which mainly involved understanding and communication. In contrast, the psychomotor component of examination skills was better in DOAP than in VAL. This indicates that while VAL can be helpful for initial learning and understanding, DOAP provides a more robust framework for retaining practical skills over time.

A study by Shirly Kurian and Gokul found that retention was better in VAL than in clinical demonstration, which was in contrast to our study. In contrast, a study by Roshini and Andrews comparing VAL versus demonstration for the mechanism of labor found that demonstration is superior to VAL, a result similar to ours. The author also emphasized in the study that, for practical skill learning, a sense of touch is essential, and this cannot be imparted through videos, though they might be interesting [15,17].

When comparing the retention of skills among those who were exposed to DOAP, we found that the mean scores of the second assessment were comparatively less than the first, indicating a slight decrease in performance over time, suggesting that immediate retention was better than long-term retention for the skills, especially those taught in sessions I, III, and VI, which mainly involve communication and interpretation. Students' performance remained relatively stable over time for the skills taught in sessions II, IV, and V, which involved skill demonstrations, suggesting better retention for these skills. The differences in results across sessions may reflect that the students were still learning. As they gained more experience and practice with the technique, their skills improved in a few areas, which might have led to higher scores in the IV session of the second assessment. Overall, the skills taught through DOAP were better retained at two weeks, particularly the demonstration aspect, with little effect on understanding and communication. These results were similar to those of Shirly Kurian and Gokul's study, which also found that demonstration was practical for immediate skill acquisition, with varied results for skill retention [15].

Similarly, assessing the retention of skills among students exposed to VAL also found a significant decrease in scores in the second assessment, indicating a notable drop in performance over time and suggesting that immediate learning was better than long-term retention for the skills taught in almost all VAL sessions. This result aligns with Shirly Kurian and Gokul's findings, showing that while VAL can be effective for immediate skill acquisition, its effectiveness tends to decrease over time. This is evident from the decreases in scores for several sessions from the first to the second assessment, highlighting a potential issue with longer-term retention when using VAL [15].

Overall, students' perception strongly favors DOAP over VAL as a teaching-learning method for obstetric examination skills in our study. DOAP is perceived as more satisfying, effective, confidence-boosting, recommendable, and engaging. Shirly Kurian and Gokul's study indicated that video-assisted teaching can be effective but may not always surpass traditional bedside demonstrations in terms of satisfaction and engagement. Our data align with this, showing higher satisfaction with DOAP [15].

Madavan also demonstrated that DOAP can significantly improve skill reproducibility through its hands-on approach, whereas VAL showed mixed results. This supports our findings that DOAP is perceived to aid reproducibility more effectively (66.67%) [12]. Shirly Kurian and Gokul's study found that while video-assisted teaching can enhance learning, it is not as effective as demonstration for retaining skills such as obstetric palpation, corroborating our results, in which DOAP is seen as more effective (73.33%) [15].

Madavan also reported that DOAP methods significantly boost confidence in performing advanced skills compared to video-assisted methods, aligning with our findings that DOAP is superior in this aspect (80%) [16]. Roshini and Andrews suggested that, despite the advantages of VAL, DOAPs are more likely to be recommended due to their effectiveness and engagement, a trend reflected in our data (73.33%) [17].

Various other studies also found that students often find DOAP more engaging than video-based methods, consistent with our findings (60%) [12,17]. This suggests that hands-on, interactive teaching methods like DOAP are more effective for learning obstetric clinical skills.

Limitations of the study

Excluding students absent on the day of teaching or assessment may introduce attrition bias into the study. With 60 students (30 in each group), the sample size is relatively small; larger samples are needed to confirm the results and ensure generalizability. The crossover design helped reduce variability between groups. Still, the possibility of a carryover effect cannot be entirely excluded, as the skills gained during the first phase may have influenced performance in the second phase, independent of the teaching method. Although the study assessed skills after two weeks, longer follow-up periods would yield more comprehensive data on the long-term retention of skills learned through DOAP and VAL.

## Conclusions

Our study shows that both DOAP and VAL methods effectively enhance the obstetric examination skills of phase 3 MBBS students. However, the DOAP method resulted in significant improvements in skill performance, retention, and confidence levels compared to the VAL method, aligning with our objective of identifying the more effective teaching strategy. In DOAP, a hands-on approach and immediate feedback from teaching faculty create a conducive environment for developing and retaining practical skills. In contrast, while VAL offers the advantages of flexibility and visual reinforcement, it may not fully replicate the interactive and practical experiences provided by DOAP.

Therefore, while integrating VAL into the curriculum can be beneficial for knowledge acquisition, we should prioritize DOAP for skill-intensive training. Combining both methods may offer a balanced approach to optimize the learning outcomes for phase 3 MBBS students in obstetric examination skills. We can design a blended learning curriculum that combines DOAP and VAL to provide comprehensive learning experiences, balancing practical, hands-on training with flexible, VAL. We should encourage faculty to be trained to adapt these methods, such as DOAP, to effectively deliver and emphasize the importance of demonstration, observation, assistance, and performance, and to create engaging and informative video content that enhances the quality and effectiveness of VAL materials.

## Appendices

**Table 5:** Feedback from the students

Sl. no	Questions	Strongly agree	Agree	Neutral	Disagree	Strongly disagree
1.	Satisfied with this teaching-learning method for antenatal examination					
2.	This method helps reproducibility					
3.	This method is more effective in helping you learn obstetric examination techniques					
4.	This method helps improve your confidence in performing obstetric examinations					
5.	Recommend this method to healthcare professionals for learning antenatal obstetric examination techniques					
6.	This is more engaging and easier to follow					
